# Disability Focal Point Persons and Policy Implementation Across Sectors: A Qualitative Examination of Stakeholder Perspectives in Zambia

**DOI:** 10.3389/fpubh.2020.00496

**Published:** 2020-09-15

**Authors:** Shaun Cleaver, Matthew Hunt, Virginia Bond, Raphael Lencucha

**Affiliations:** ^1^School of Physical and Occupational Therapy, McGill University, Montreal, QC, Canada; ^2^Centre for Interdisciplinary Research in Rehabilitation of Greater Montréal (CRIR), Montreal, QC, Canada; ^3^Zambart, School of Public Health, University of Zambia, Lusaka, Zambia; ^4^Department of Global Health and Development, Faculty of Public Health and Policy, London School of Hygiene and Tropical Medicine, London, United Kingdom

**Keywords:** coordination, disability, focal point, mainstreaming, representation, whole-of-government approach, Zambia

## Abstract

**Background:** Zambia has created new disability policies and updated existing policies to be consistent with the United Nations Convention on the Rights of Persons with Disabilities. These initiatives require the widespread engagement of ministries and departments to achieve effective policy development and implementation. To pursue widespread engagement, the Government of Zambia developed a structure of disability focal point persons (FPPs). The Zambian disability FPP structure has not yet been explored systematically.

**Objective:** To explore disability policy stakeholder perspectives about FPPs as a feature of disability policy development and implementation.

**Methods:** We conducted semi-structured interviews with 24 disability policy stakeholders (10 policymakers, 2 researchers, and 12 disability advocates) as part of a larger study about the development and implementation of disability-related policies in Zambia. Interviews were audio recorded and transcribed. Data were analyzed using content analysis.

**Results:** Participants presented FPPs as a promising way to mainstream disability within the government. According to participants, the initial launch of the FPP structure was ineffective, with a lack of clarity about the structure and an initial cohort of FPPs that wielded minimal influence. The FPP structure has since been revised. Participants express promise that the improved second launch will achieve mainstreaming.

**Discussion:** Zambian disability policy stakeholders describe a disability FPP structure that is different from the models suggested for treaty implementation. Pre-established commitments to mainstreaming among stakeholders might have stimulated interest in following the cyclical development of the disability FPP structure, encouraging a whole-of-government approach to disability policy implementation.

## Introduction

Adults and children with disabilities face multiple forms of disadvantage that negatively affect their quality of life and health outcomes (1). Progressive disability policy is an important strategy to address these disadvantages. The international landscape of progressive disability policy changed markedly with the adoption of the United Nations Convention on the Rights of Persons with Disabilities (2). States party to the CRPD commit to reviewing and modifying their domestic policies to fulfill the provisions of the Treaty (3). After having signed and ratified the CRPD in 2008 and 2010, respectively, Zambia has since made major policy changes through a revision of its keystone disability legislation (4) and the creation of an inaugural national disability policy (5). As Zambia is a unitary republic, the central government is responsible for policymaking while the role of provinces and districts is limited to implementation.

While the development of new policies is an important achievement, implementation throughout a government's activities remains a critical and ongoing challenge. The need for coordinated implementation, a “whole-of-government” approach (6), has been recognized in administrative circles. Within disability advocacy circles, the similar idea of incorporating disability issues across government ministries, departments, and agencies (e.g., Information & Communications Technology, Anti-Corruption) is often referred to as “mainstreaming” (7). According to the principles of mainstreaming, every part of the government must engage in disability issues.

Article 33 of the CRPD provides direction to countries on implementation. This article, titled “National implementation and monitoring,” stipulates that “States Parties, in accordance with their system of organization, shall designate one or more focal points within government for matters relating to the implementation of the present Convention” (2). While the need for focal points is outlined in the CRPD, this mechanism is nearly absent from Zambia's primary policy documents: there are no references to focal points in the Persons with Disabilities Act (4) and a lone reference to focal point *persons* in the implementation plan of objective 5 of the National Policy on Disability (5).

Despite the relative silence on the issue of focal points in Zambia's primary disability policy documents, there is reason to believe that focal point persons (FPPs) are of interest within the country. In a case study about disability related policymaking, it is noted that “Zambia initially chose to designate several focal persons in the relevant Ministries under article 33(1) of the CRPD to coordinate the implementation of the Convention” (8). Unfortunately, there is a lack of publicly available information from the government to confirm the details of this mechanism and confirm that its purpose is to satisfy the terms of Article 33. The Zambia Agency for Persons with Disabilities (ZAPD), a unit within the Ministry of Community Development and Social Services, is the government agency responsible for disability. ZAPD's current strategic plan refers to FPPs, mentioning them 23 times, often as part of the outputs and activities associated with their strategic outcomes (9). While the strategic plan does not make explicit links between FPPs and the CRPD, in a report about Zambia's implementation of the CRPD from the perspective of disability advocates, the “creation of disability focal point persons” is identified as one of five “Efforts made by the government of Zambia to domesticate the UNCRPD” (10). The remaining four efforts were major policies, illustrating that FFPs were seen to be a priority policy consideration.

It is generally expected that there should be consistency among the CRPD, Zambia's main disability policy documents, ZAPD's strategic direction, and the interests of the disability advocacy community (11). The subject of focal points presents notable inconsistencies. Whereas, the CRPD's Article 33 states that there must be focal points—which could be institutions or persons—domestic Zambian documents consistently refer only to focal point *persons*. As established above, there is but one reference to focal point persons within Zambia's main disability policy documents. This relative silence is contrary to reports about CRPD implementation that emphasize their importance (8, 10) and ZAPD's frequent references in its strategic plan (9). According to available documentation, the nature and the importance of focal points for Zambian disability policy varies widely.

While there are references to FPPs in Zambia, details about their structure and function are lacking. Without specifying the time period or citing a reference, Zimba claims that ten Zambian government ministries and three agencies had disability FPPs (10). ZAPD planned that “the Focal Point Persons system and structure (was) to (be) decentralized” (9). The agency did not define “decentralized” but presented plans to have FPPs in government line ministries, provinces, districts, and churches (9). These plans are definitely well beyond the CRPD's stipulation that there be “one or more focal points within government” (2). As the strategic plan was forward-looking, the document offered only minimal description of the situation and evolution of disability FPPs. Nonetheless, the agency identified the threat of, “inadequate understanding of disability issues and the policy and legislative framework among the Government FPPs” (9). Moreover, the agency's plans suggestively state that there is a need for “building capacity of the FPPs to actually undertake their roles and responsibilities” and identify the assumption that service delivery coordination requires that “the right people are selected as FPPs” (9).

Zambia's primary disability policies require coordinated implementation across government sectors. Disability FPPs could be a valuable mechanism to achieve this widespread involvement and facilitate effective policy implementation. Despite this promise, there are conflicting indicators about the importance of FPPs and only minimal, yet suggestive, details about their role thus far. There is therefore a need to illuminate the roles and realities of disability FPPs in Zambia. The purpose of this analysis is therefore to explore disability policy stakeholder perspectives about FPPs that were expressed during interviews about the Zambian disability policy development process.

## Methods

We used a qualitative descriptive design (12) as part of a larger research project investigating the development of disability-related policies in Zambia. Qualitative description allows flexibility in data collection and inductive analysis that, “entails a kind of interpretation that is low-inference, or likely to result in easier consensus among researchers” (12). Policy development was conceptualized according to the four phases of the policy cycle as described by Jack (13): problem identification, policy formulation, implementation, and evaluation. Twenty-four disability policy stakeholders (10 policymakers, 2 researchers, and 12 disability advocates) participated in the research. The demographics of participants are presented in Table 1. The sample size of 24 participants was established with a vision to have eight participants in each stakeholder category. During the recruitment phase, a surplus of policymakers and disability advocates volunteered to participate while fewer researcher participants could be located.

**Table 1:** Demographic description of participating disability policy stakeholders.

**Participant characteristic**	**Disability advocates**	**Policymakers**	**Researchers**
**Gender**			
Women	4	6	1
Men	8	4	1
**Self-identifies as disabled**			
Yes	9	0	0
No	3	10	2
Totals	12	10	2

### Data Collection and Analysis

Data for this study were collected in two fieldwork phases (in 2018 and 2019) through 27 individual interviews (three participants were interviewed twice). The interviews were semi-structured, lasting 30–90 min. Interview content focused on the participants' own experiences engaging with the policy process in the development of Zambian disability policies. Participants were also asked to describe the structures, relationships, and processes (14) that guided the development of the policies with which they had engaged.

The FPP topic emerged spontaneously in multiple interviews without specific prompts, questions, or the interviewer having a thorough understanding of focal points at the time of the interviews. Interviews were audio recorded and transcribed. Data were initially coded for all references to FPPs. We then analyzed these references using content analysis (15).

### Ethical Considerations

This study was approved by McGill University, Faculty of Medicine, Institutional Review Board Institutional Research Board (Protocol reference #: A12-B68-17B), the University of Zambia Biomedical Research Ethics Committee (Protocol reference #: 011-01-18), and the Zambia National Health Research Authority. Informed consent was provided by all participants prior to beginning the interview. All data were anonymized during the transcription process and securely stored on encrypted media.

## Results

Twelve of 24 participants spoke about FPPs in their interviews about their involvement in the disability policy process. These 12 participants are presented in more detail in Table 2, including the number of times that they referred to FPPs. In general, participants spoke about two main themes in relation to FPPs: (1) disability FPPs constitute a promising structure to mainstream disability within the government and (2) the implementation of the initial FPP structure was ineffective. An early version of the content analysis codes is presented in Table 3 while each of these themes is described in detail in the text.

**Table 2:** Individual participants who referred to focal point persons.

**Participant number**	**Stakeholder category**	**Self-identifies as disabled**	**Self-declared disability type**	**Nb. of interviews**	**Nb. of references**
1	Advocate	Yes	Hard of hearing	1	4
2	Policymaker	No	NA	2	59
3	Advocate	Yes	Blind	1	1
4	Advocate	Yes	Physical	1	36
5	Advocate	No	NA	2	7
6	Policymaker	No	NA	1	7
7	Policymaker	No	NA	1	7
8	Policymaker	No	NA	1	4
9	Policymaker	No	NA	1	2
10	Policymaker	No	NA	1	3
11	Policymaker	No	NA	1	3
12	Researcher	No	NA	1	6

*NA, not applicable*.

**Table 3:** Early codes developed through content analysis.

**Code name**
Ad hoc FPP selection
Belief in the potential of FPPs
Mainstreaming
Low-level FPPs
Lack of direction for FPPs (no terms of reference; no training)
FPPs not empowered to build a collective structure
High performing FPPs in ministries active in disability issues
Insufficient instructions for ministries

### Disability FPPs Constitute a Promising Structure to Mainstream Disability Within the Government

Participants consistently discussed the evolution of the disability FPP structure according to three stages: the launch of the disability FPP structure within ministries, the initial implementation of this structure, and the revision of the structure in anticipation of a second launch. The preparatory processes for the second launch, including the recruitment and training of new FPPs, were still ongoing at the time of the final interviews in April 2019. Meanwhile, the timeline for the entirety of the second launch had not yet been clearly established. Only one policymaker with extensive involvement in disability issues, participant number 2, offered some chronological insight by stating that the stakeholders responsible for the FPP structure “started to push” for a revision of the initial structure in late 2017.

Participants broadly identified the disability FPP structure as one with important potential to promote disability mainstreaming within the Government of Zambia's line ministries. According to participant 2, “the Zambia Agency for Persons with Disabilities and the Ministry of Community Development are all looking forward to the issue of mainstreaming to be done through the focal point persons.”

One policymaker described FPPs as “inter-sitters in government who will be like the key persons in every ministry” (participant 6). Multiple participants described the FPP structure, both the initial and revised versions, as consisting of a single point person in each line ministry. “Each ministry should send one person, or should choose one person, who should just be the center person where disability issues are concerned” (participant 4). No participants discussed the possibility of FPPs being present in other governmental institutions such as agencies, or as liaisons between government bodies, civil society, or the private sector.

Participants presented three general characterizations of the overall role of FPPs, though they did not discuss the intensity of the FPP role (e.g., the amount of time a disability FPP would be expected to devote to this role).

#### Characterization 1: FPPs as Directors

The first characterization was that the FPP should operate like a director of disability issues within their respective ministry. According to this characterization, FPPs would be responsible for initiating and managing programs within their respective ministry. Supporting this characterization, one disability advocate stated, “we want to see that those focal persons, in those ministries, they should also understand and embrace the roles which they will be given by government, and also have their budget line to support programs” (participant 5).

#### Characterization 2: FPPs as Coordinators

A second characterization was that FPPs were to function as coordinators, acting as a bridge between parties in order to facilitate the flow of information and ensure that certain perspectives are shared. Policymakers who were closest to the development of the FPP structure discussed it according to this characterization. One of these policymakers (participant 2) stated that:

…one thing that we will need to have from the focal point persons are reports, like what is being done concerning the mainstreaming. Because, when we are talking about a particular program (their ministry is) doing which ensures that disabilities are included, if it's budgeting, the way budgeting for 2019, (the FPP) ensures that the component of disability was put in place.

According to this second characterization, the flow of information between parties would ensure that disability issues were addressed in the ministries with disability FPPs.

#### Characterization 3: FPPs as Front-Line Workers

The third characterization of the role of FPPs was that FPPs should be front-line workers. One disability advocate, participant 1, described FPPs in a manner which initially seemed to be consistent with the characterization of FPPs as coordinators, but his description was ultimately more of a front-line role. According to participant 1, “(the Ministry of Health) can still do much better. Especially if they had a disability focal point person who is able to coordinate all types of disability.” This participant continued to describe challenges for persons with disabilities to access programs and services, proposing that, “there should be somebody who is supposed to help a person who is blind, even a person who is on a wheelchair.” In this account, the participant did not specify that it was the ministry's FPP who should offer this support directly. Nonetheless, the participant followed his proposals with the statement that, “(the ministry is) still not doing much in all areas because of lacking a disability focal point person.”

The characterization of FPPs as front-line workers was exceptional among stakeholders participating in the study; participant 1 was the only one who presented an account where disability FPPs might be understood according to this characterization. However, another disability advocate (participant 4) expressed the belief that that this characterization was widely held within Zambian line ministries, “for them, they think to be a focal point person, it's like when a person with disability comes to the ministry, you help them maybe go to the loos (toilet) and so on.” From the perspective of participant 4 this view was a common yet erroneous understanding of the FPP role that undermined the potential of FPPs to enable broad operational changes.

Regardless of the characterization they offered for the role of the FPPs, participants expressed substantial confidence that the FPP structure would effectively transform the extent to which disability was mainstreamed within the Government of Zambia.

### The Initial Implementation of the FPP Structure Was Ineffective

Despite the promise of the FPP role to enhance mainstreaming, participants identified that the initial implementation of the disability FPP structure was ineffective. Participants proposed multiple explanations for the ineffective implementation, particularly a lack of clarity with respect to the expectations of ministries and individual FPPs. In discussing the initial implementation, participants identified two interrelated problems which are described below: the roll out was conducted without first clarifying the nature of the FPP structure and the process of selecting FPPs was flawed. At the time when the interviews were being conducted, a reformulation of the FPP structure was underway. Participants expressed optimism that this revised mechanism would effectively mainstream disability issues throughout the Government of Zambia.

#### Lack of Clarity Around the FPP Structure in the Initial Implementation

One disability advocate encapsulated the lack of clarity during the initial implementation, stating, “I think we have not understood much what that focal point person means” (participant 4). Along with this statement, she contrasted the initial misunderstanding within Zambian line ministries to improvements with the situation prior to the second launch: “Although the understanding and, like their role exactly, was not… even the focal point persons were not very sure. After the (Persons with Disabilities) Act, now we understand very well, both, we advocates and government.”

While all participants expressed accounts that were consistent to participant 4's description of disorganization followed by improvement, not all agreed on the timeline or the sequence of events. The Persons with Disabilities Act came into force in 2012 (4). Meanwhile, according to one policymaker involved in revising the disability FPP structure (participant 7), “the first time I came across it was in 2016 when they were talking about, “you know the focal point persons, nothing is working.” I said, “okay, show me their terms of reference.” Nobody had terms of reference. So how were they going to work?”

#### Initial Cohort of Disability FPPs Unable to Influence Ministries

With a lack of clarity around the FPP structure, it might be understandable that the initial selection of FPPs was not conducted in a strategic and consistent manner across ministries. For the participants, the largest problem with the initial selection of FPPs was that these were ministry employees in low level positions who were unable to access ministry information or influence decisions. Participant 2, a policymaker who had been responsible for collaborating with the initial cohort of FPPs described the situation as follows: “When making follow ups to get reports from them, it was difficult to get what has been done. Even if you talk to them, you write to them, it was kind of difficult for them to go and report back and ensure that those initiatives were implemented.” Participant 2 explained this dynamic through the selection process:

Early on, there was a circular that was circulating in government ministries to ensure that focal point persons were appointed, but it's just sad to note that at that time, people that were being appointed were not those in decision making positions. You would find someone who is just in an administrative office.

Participant 2's observations from a policymaker position were corroborated and further explained by a disability advocate:

Participant 4: Some ministries have (identified FPPs), but the kind of people that they pick to be focal points persons, you know, their positions are not those positions whereby they can influence. Even when they go to meetings, they cannot make decisions and when they go back, they cannot even influence any, any decision.

Interviewer: Sort of low-level people.

Participant 4: It's low people, their positions are very, very low. In fact, in some ministries if they have someone with a disability, they will pick on that one, regardless of the qualification or the position.

Interviewer: If it is a cleaner, a receptionist…

Participant 4: Yes.

### Common Considerations in the Two Themes

Participants expressed that there was ineffective initial implementation while simultaneously describing the significant promise of the FPP structure. These two points might appear contradictory, until one considers the thorough explanations of the initial failure and the associated revisions made to the FPP structure as part of the second launch.

In effect, these two themes support a common idea that the FPP structure will mainstream disability, a conviction which was reinforced rather than undermined by the initial implementation. While thought to be unsuccessful, the initial implementation was attributed to a limited number of problems that were considered to now be resolved. Throughout these accounts, the faith in the effectiveness of the FPP structure and in mainstreaming was strong.

## Discussion

Among Zambian disability policy stakeholders participating in this study, there was frequent and passionate discussion about FPPs. The FPP structure was perceived to be a transformative mechanism to mainstream disability issues throughout the Government of Zambia's activities. Participants had a shared understanding that the initial implementation of the FPP structure was ineffective at achieving the desired goal of mainstreaming disability throughout government. In explaining the details and the causes of the ineffective initial launch, participants never expressed doubt about the potential of the FPP structure nor about the need for disability mainstreaming.

The participants' descriptions of the disability FPP structure in Zambia differ from the international models suggested for focal points to support CRPD implementation and the guidance provided about focal points for other international treaties. First, participants universally described focal points *as persons*. There is reason to believe that this personification of disability focal points is longstanding in Zambia, given that focal points are also presented this way in Zambian government documents and publications.

There are arrangements in which focal points are, by definition, individuals [see reference (16) for an example from a different international agreement] but this more restricted definition is not consistent with the supporting documentation of the CRPD. According to the *Handbook for Parliamentarians on the Convention on the Rights of Persons with Disabilities*, an international document created to inform national legislatures of details relevant to the CRPD, “Focal points could be a section or a person within a ministry or cluster of ministries, an institution, such as a disability commission, or a particular ministry, such as a ministry for human rights or a ministry for persons with disabilities, or a combination of the three” (17). Examples of institutional focal points include the Directorate-General structure implemented by Italy (18) and the tri-partite structure implemented by Finland (19).

Like the personification of focal points, the participants' description of the roles of disability FPPs was a subset of those which have been suggested. Participants consistently described the FPP structure as one that was created to mainstream disability within the Government of Zambia. Meanwhile, the *Handbook for Parliamentarians* suggests 16 actions that could be the work of focal points (see Table 4). Of these 16 actions, six could be consistent with mainstreaming activities (i.e., those related to intra-governmental coordination, raising awareness, building capacity, and collecting data). By contrast, most of the 16 suggested actions have goals that are different than mainstreaming and would not be well-addressed by the “one FPP per line ministry” structure that participants described.

**Table 4:** The work of focal point persons [As presented in the *Handbook for Parliamentarians* (17)].

• Advise the Head of State/Government, policymakers and programme planners on the development of policies, legislation, programmes and projects with respect to their impact on people with disabilities;
• Coordinate the activities of various ministries and departments on human rights and disability;
• Coordinate activities on human rights and disability at federal, national, regional, state, provincial and local levels of government;
• Revise strategies and policies to ensure that the rights of persons with disabilities are respected;
• Draft, revise or amend relevant legislation;
• Raise awareness about the Convention and Optional Protocol within the Government;
• Ensure that the Convention and Optional Protocol are translated into local languages and issued in accessible formats;
• Establish an action plan for ratification of the Convention;
• Establish an action plan for implementation of the Convention;
• Monitor the implementation of the action plan on human rights and disabilities;
• Coordinate the preparation of the State's periodic reports;
• Raise awareness on disability-related issues and the rights of persons with disabilities among the public;
• Build capacity within the Government on disability-related issues;
• Ensure and coordinate the collection of data and statistics for effective policy programming and evaluation of implementation;
• Ensure that persons with disabilities participate in the development of policies and laws that affect them;
• Encourage persons with disabilities to participate in organizations and civil society, and encourage the creation of organizations of persons with disabilities.

Beyond disability policy, a number of guidance documents published as compendiums to international treaties outline the role of focal points, demonstrating a need to clarify their roles [e.g., (16, 20)]. These documents present a wide variety of activities that focal points can pursue. Nonetheless, the clearest mandate that is consistently found in such documents is the role of focal points as conduits of information between international and national governing bodies, a role that was never expressed by participants.

The semi-structured interviews upon which this study is based were designed to explore Zambian disability policy development in a broad sense. For this reason, participants were not probed about the difference between the Zambian disability FPP structure and the international guidance. Nor were participants asked about their awareness of that international guidance. Despite the lack of data on these issues, the participants did provide significant information about their interest in mainstreaming disability throughout the government's activities. Given that interest, it is understandable that there was broad support for a disability FPP structure that privileged mainstreaming roles, even if this structure might neglect the other concerns outlined in guidance documents.

Although the FPP structure was not yet effective at the time of the research and a second launch was underway, the perspectives of disability policy stakeholders on FPPs illuminate phenomena that could be of interest to a wider health policy community. Disability is often considered to be an issue of marginal relevance in the policy domain, despite the fact that nearly everyone is touched by disability at some point in their lives (21). Zambian disability policy stakeholders face the challenge of designing robust and effective policies—a challenge that is universal for those involved in policy development—along with the supplementary challenges of working from the position of a marginalized issue. Whereas, whole-of-government approaches (6) might seem daunting or overly complicated in some policy spheres, among the stakeholders interviewed in this study, there was enthusiastic support for this approach as a means of achieving disability mainstreaming. This analysis provides a first step into understanding the role of FPPs in this mainstreaming process specifically, and as a mechanism for whole-of-government policy implementation generally.

It is possible that there are contextual factors that contribute to the support for a whole-of-government approach among disability policy stakeholders in Zambia. Most importantly, disability policy has developed globally because of and alongside a movement to reform understandings of disability and the place of persons with disabilities (21). This disability movement was a direct response to patterns of segregation and exclusion, such that it is logical that this movement is organized around demands for inclusion (22). The disability movement was already well-advanced in terms of both scholarship and advocacy when the concept of mainstreaming was first articulated in 2000 in the context of disability inclusive development (7). In this context, mainstreaming, “entails the inclusion of an active consideration of disability issues in the mainstream of development co-operation work” (7). The concept of mainstreaming has since been taken up more widely in disability circles, as applied to education (23), healthcare (24), and public policy (25), among others. Mainstreaming is typically presented as one of the two elements of the “twin track approach.” The other element is disability-specific programming, which is presented as a complementary alternative that can be more appropriate in certain circumstances where mainstreaming is not possible.

## Conclusion

The results of this analysis draw attention to a national implementation process of the CRPD and generate insights about approaches to public policy implementation more broadly. Participants in this research spoke of the Zambian FPP structure—its promise and its ineffectual initial implementation—while simultaneously expressing their faith in the value of mainstreaming disability in all aspects of government.

Policymakers can have difficulty implementing a whole-of-government approach (6) and maintaining stakeholder attention through all stages of the policy cycle (13). In seeking solutions to overcome these difficulties, we recommend that policymakers take note of the situation of disability FPPs in Zambia as an example of a policy stakeholder community where a whole-of-government approach is intuitive and engagement is high. Despite the challenges of implementing the disability FPP structure in Zambia, the widespread commitment to mainstreaming disability seems to facilitate alignment between policymakers and other stakeholders, demonstrating that policymakers can garner more support when aligning with community perspectives (14).

## Data Availability Statement

The datasets generated in this study are interview transcripts that cannot be anonymized sufficiently to ensure participant confidentiality. Inquiries pertaining to data and data quality can be directed to the authors. The terms of research ethics approval stipulate that the authors must store data for a minimum of seven years after publication.

## Ethics Statement

The studies involving human participants were reviewed and approved by McGill University, Faculty of Medicine, Institutional Review Board, the University of Zambia Biomedical Research Ethics Committee, and the Zambia National Health Research Authority. Written informed consent to participate in this study was provided by the participants' legal guardian/next of kin.

## Author Contributions

SC designed and implemented the larger study, of which this analysis is a subset, with the guidance of MH, VB, and RL. RL provided additional policy expertise that supported the framing of the analysis and identified influential literature on focal point structures. SC drafted the core article. RL drafted a number of sub-sections within the article. All authors contributed intellectual content to the project and approved the final manuscript prior to submission.

## Conflict of Interest

The authors declare that the research was conducted in the absence of any commercial or financial relationships that could be construed as a potential conflict of interest.
